# Afatinib induces apoptosis in NSCLC without EGFR mutation through Elk-1-mediated suppression of CIP2A

**DOI:** 10.18632/oncotarget.2941

**Published:** 2014-12-11

**Authors:** Ting-Ting Chao, Cheng-Yi Wang, Yen-Lin Chen, Chih-Cheng Lai, Fang-Yu Chang, Yi-Ting Tsai, Chung-Hao H. Chao, Chung-Wai Shiau, Yuh-Chin T. Huang, Chong-Jen Yu, Kuen-Feng Chen

**Affiliations:** ^1^ Medical Research Center, Cardinal Tien Hospital, School of Medicine, Fu Jen Catholic University, New Taipei City, Taiwan; ^2^ Department of Internal Medicine, Cardinal Tien Hospital, School of Medicine, Fu Jen Catholic University, New Taipei City, Taiwan; ^3^ Graduate Institute of Clinical Medicine, College of Medicine, National Taiwan University, Taipei, Taiwan; ^4^ Department of Pathology, Cardinal Tien Hospital, School of Medicine, Fu Jen Catholic University, New Taipei City, Taiwan; ^5^ Department of Intensive Care Medicine, Chi Mei Medical Center, Liouying, Tainan, Taiwan; ^6^ Instrumentation Resource Center, National Yang-Ming University, Taipei, Taiwan; ^7^ Institute of Biopharmaceutical Sciences, National Yang-Ming University, Taipei, Taiwan; ^8^ Department of Medicine, Duke University Medical Center, Durham, North Carolina, USA; ^9^ Department of Internal Medicine, National Taiwan University Hospital and National Taiwan University, Taipei, Taiwan; ^10^ Department of Medical Research, National Taiwan University Hospital, Taipei, Taiwan; ^11^ National Center of Excellence for Clinical Trial and Research, National Taiwan University Hospital, Taipei, Taiwan

**Keywords:** CIP2A, EGFR, PP2A, Elk-1, afatinib

## Abstract

Afatinib has anti-tumor effect in non-small cell lung carcinoma (NSCLC) with epidermal growth factor receptor (EGFR) mutation. We found afatinib can also induce apoptosis in NSCLC cells without EGFR mutation through CIP2A pathway. Four NSCLC cell lines (H358 H441 H460 and A549) were treated with afatinib to determine their sensitivity to afatinib-induced cell death and apoptosis. The effects of CIP2A on afatinib-induced apoptosis were confirmed by overexpression and knockdown of CIP2A expression in the sensitive and resistant cells, respectively. Reduction of Elk-1 binding to the CIP2A promoter and suppression of CIP2A transcription were analyzed. *In vivo* efficacy of afatinib against H358 and H460 xenografts tumors were also determined in nude mice. Afatinib induced significant cell death and apoptosis in H358 and H441 cells, but not in H460 or A549 cells. The apoptotic effect of afatinib in sensitive cells was associated with downregulation of CIP2A, promotion of PP2A activity and decrease in AKT phosphorylation. Afatinib suppressed CIP2A at the gene transcription level by reducing the promoter binding activity of Elk-1. Clinical samples showed that higher CIP2A expression predicted a poor prognosis and Elk-1 and CIP2A expressions were highly correlated. In conclusion, afatinib induces apoptosis in NSCLC without EGFR mutations through Elk-1/CIP2A/PP2A/AKT pathway.

## INTRODUCTION

Lung cancer is the leading cause of cancer-related deaths worldwide [1]. It is broadly classiﬁed into two major categories: small cell lung cancer and non-small cell lung cancer (NSCLC), and 80% of lung cancers are NSCLC [1]. Platinum-based chemotherapy is currently the standard treatment for NSCLC [2-4], but targeted therapies with few side effects may compensate for the incompleteness of conventional chemotherapy. Epidermal growth factor receptor (EGFR) gene mutations have been reported in 10-15% of Caucasian patients with NSCLC, and in an even higher percentage of Asian patients [5]. NSCLC patients with certain EGFR mutations such as L858R and exon 19 deletion have been reported to have a higher response rate to the EGFR tyrosine kinase inhibitors (TKIs) than those without EGFR-activating mutations [6-9]. However, some NSCLC patients without EGFR mutation still respond to TKIs [6-11], which suggesting that there may be mechanism(s) other than the EGFR pathway by which TKIs can cause apoptosis in NSCLC cells without EGFR mutation. Elucidating this mechanism may offer a new therapeutic option for NSCLC patients without EGFR mutation.

Cancerous inhibitor of protein phosphatase 2A (CIP2A) was originally identified as a cellular PP2A inhibitor that inhibits the proteolytic degradation of c-MYC [12, 13]. CIP2A has been found to be overexpressed in several human malignancies including hepatocellular carcinoma, gastric cancer, head and neck cancer, colon cancer, breast cancer, prostate cancer and NSCLC [12-21]. In addition, CIP2A is overexpressed in NSCLC and correlated with a poor prognosis [12, 15, 18, 21], and the downregulation of CIP2A and inactivation of the AKT pathway has been reported to inhibit proliferation and induce apoptosis in a variety of lung cancer cells [12, 18].

Afatinib is new generation TKI, and is an irreversible inhibitor of the tyrosine kinase activity of members of the epidermal growth factor receptor family (ErbB) including EGFR, HER2 and ErbB4 [12, 18, 22, 23]. Afatinib covalently binds to cysteine 797 of EGFR and cysteines 805 and 803 in HER2 and ErbB4, respectively. Such covalent binding irreversibly inhibits the tyrosine kinase activity of these receptors, resulting in reduced auto- and trans-phosphorylation within the ErbB dimers and inhibition of important steps in the signal transduction of all ErbB receptor family members. Afatinib has anti-tumor effect in NSCLC with EGFR mutations [24-26]. Afatinib has anti-tumor effect in NSCLC with activating EGFR mutations, approved by EU, US FDA and Taiwan FDA [22-27]. However, we found afatinib also induced apoptosis in NSCLC cell lines without EGFR mutation. The aim of this study was to investigate the mechanistic basis for the effect of afatinib in NSCLC without EGFR mutations.

## RESULTS

### Differential effects of afatinib on cellular apoptosis in the NSCLC cell lines

**Figure 1:**
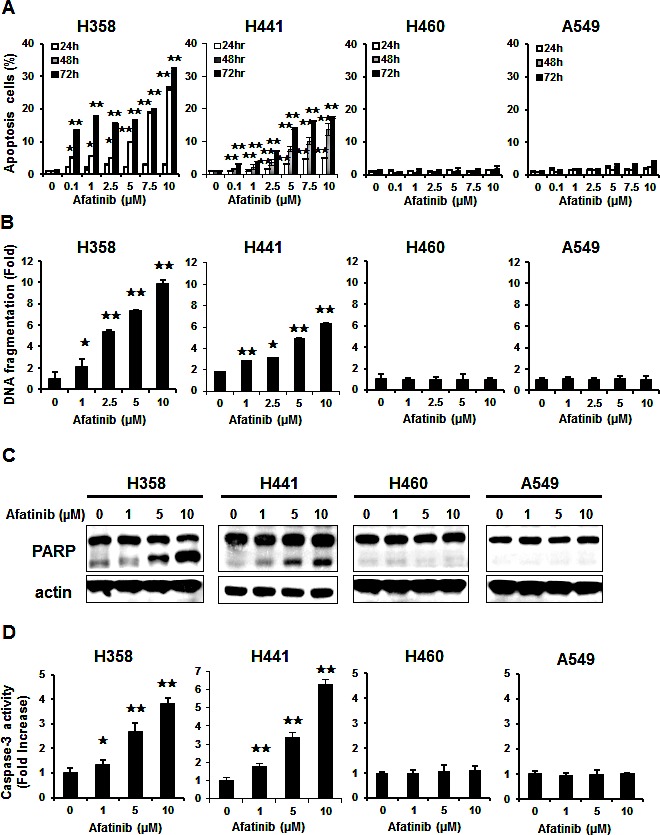
Differential effects of afatinib on cell apoptosis and death in the four human NSCLC cells (A) Dose-dependent effects of afatinib on apoptosis. Data are mean ± SD. n = 3 for each concentration. *, p < 0.05, **, p < 0.01, vs. no afatinib. (B) Dose-dependent effects of afatinib on DNA fragments. Data are mean ± SD. n = 3 for each concentration. *, p < 0.05, **, p < 0.01, vs. no afatinib. (C) Effects of afatinib on PARP in the four NSCLC cell lines. Cells were treated with afatinib at the indicated concentrations for 48 h. Data are representative of three independent experiments. (D) Effects of afatinib on caspase-3 activity. NSCLC cells were treated with afatinib at the indicated concentrations for 48 h. Data are mean ± SD. n = 3 for each concentration. *, p < 0.05, **, p < 0.01, vs. no afatinib.

To examine the antitumor effects of afatinib on the NSCLC cell lines without EGFR mutation, we conducted FACS analysis by flow cytometry using propidium iodide (PI) to analyze the sub-G1 percentage. As shown in Figure 1A, afatinib exhibited differential apoptotic effects on the NSCLC cell lines. The H358 and H441 cells showed significant apoptotic effects in the presence of afatinib in a dose- and time-dependent manner. In contrast, the H460 and A549 cells were less sensitive to afatinib-induced apoptosis. In addition, the apoptotic effects were further evaluated by DNA fragmentation assay, determination of caspase-3 activity, and Western blot analysis of cleaved PARP. In the H358 and H441 cells, we found that the cells exposed to afatinib had increased DNA fragmentation (Figure 1B), cleaved PARP (Figure 1C) and caspase-3 activity (Figure 1D) but not in the H460 and A549 cells. These results indicate that afatinib induces apoptosis in H358 and H441 cells.

### Downregulation of CIP2A determined afatinib–induced apoptosis through p-AKT inhibition in the NSCLC cell lines

**Figure 2:**
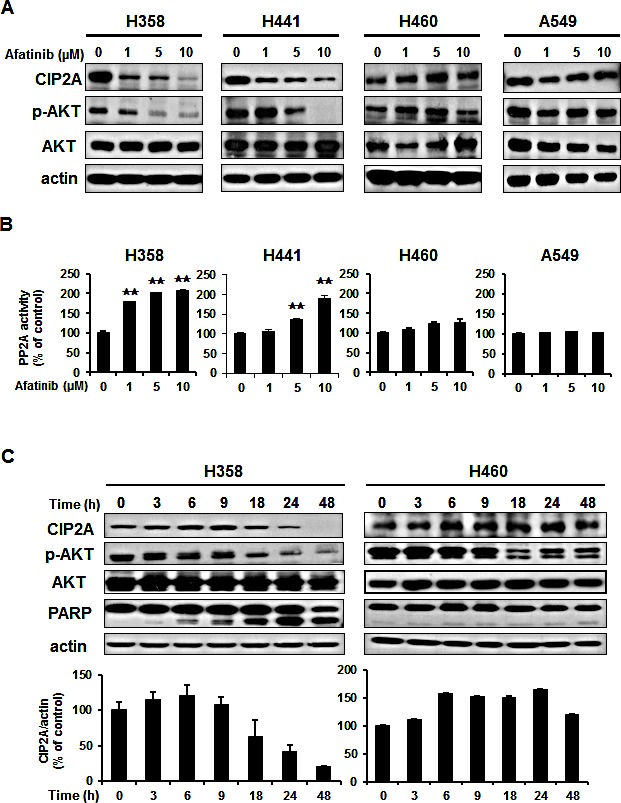
Downregulation of CIP2A determined the effects of afatinib on p-AKT and apoptosis in NSCLC cells through PP2A activation (A) Dose-dependent effects on CIP2A, p-AKT and AKT. (B) Effects of afatinib on PP2A activity. NSCLC cells were exposed to afatinib at the indicated concentrations for 24 h. Data are mean ± SD. n = 3 for each experiment. **, p < 0.01, vs. no afatinib. (C) Time-dependent effects of afatinib on CIP2A, p-AKT and apoptosis-related proteins in the sensitive H358 and resistant H460 cells. Cells were exposed to 10 μM afatinib for up to 48 hours. Immunoblots were scanned and quantitated to determine the ratio of the level of CIP2A to actin. Data are mean ± SD. n = 3 for the different time intervals.

According to the differential effects of afatinib on apoptosis (Figure1), we defined the H358 and H441 as afatininb-sensitive cell lines, and H460 and A549 as afatininb-resistant cell lines. We next investigated the role of CIP2A in afatinib-induced apoptosis in the NSCLC cells. As shown in Figure 2A, afatinib decreased CIP2A protein levels and AKT phosphorylation in the afatinib-sensitive H358 and H441 cells in a dose- and time-dependent manner (Figure 2C). In contrast, afatinib did not significantly decrease CIP2A protein levels or AKT phosphorylation in the resistant H460 and A549 cells. These results indicated that the CIP2A signaling pathway may play an important role in determining the sensitivity of lung cancer cells to afatinib. Previous studies demonstrated that CIP2A is an oncogenic PP2A inhibitor protein that is highly expression in malignant cancers. We found that the PP2A activity increased (Figure 2B) when protein level of CIP2A decreased (Figure 2A) in the sensitive H358 and H441 cells, whereas, afatinib did not change PP2A activity (Figure. 2B) or protein level of CIP2A (Figure 2A) in the resistant H460 and A549 cells. Taken together, these data indicate that the CIP2A-PP2A-AKT pathway may mediate the sensitizing effect of afatinib.

### Afatinib enhanced the NSCLC cells apoptosis and cell death through the mechanism of CIP2A-PP2A-AKT

**Figure 3:**
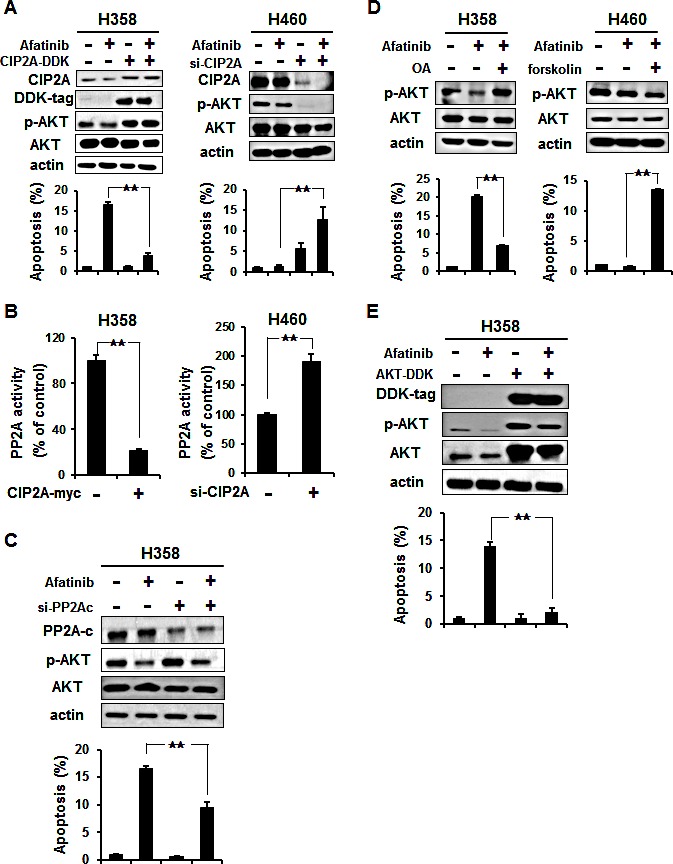
Validation of the CIP2A-PP2A-AKT pathway (A) Left, ectopic expression of CIP2A (CIP2A-DDK) increased p-AKT and attenuated the effects of afatinib on apoptosis of H358 cells. H358 cells overexpressing CIP2A were treated with 10 μM afatinib for 24 h. Right, knockdown of CIP2A expression by siRNA increased the sensitivity to afatinib-induced apoptosis in H460 cells. Cells were transfected with either control or CIP2A siRNA for 48 h then exposed to 10 μM afatinib for 24 h. (B) Left, overexpression of CIP2A decreased PP2A activity in H358 cells. Right, downregulation of CIP2A by siRNA increased the activity of PP2A in H460 cells. (C) Silencing PP2Ac reduced the apoptotic effect of afatinib in H358 cells. Cells were transfected with either control or PP2Ac siRNA for 48 h then exposed to 10 μM afatinib for 24 h. (D) Left, okadaic acid (OA), a PP2A inhibitor, increased p-AKT and inhibited the effects of afatinib on apoptosis of H358 cells. Right, forskolin, a PP2A agonist, sensitized resistant H460 cells to afatinib. Data are mean ± SD. n = 3 for each condition. **, p < 0.01, vs. no afatinib. (E) Ectopic expression of AKT (AKT-DDK) attenuated the effects of afatinib on apoptosis of H358 cells. H358 cells overexpressing AKT were treated with 10 μM afatinib for 24 h.

To verify the role of CIP2A/PP2A/p-AKT in mediating afatinib-induced apoptosis in the EGFR wild-type sensitive NSCLC cells, two approaches were used. First, the ectopic expression of CIP2A was evaluated in the sensitive H358 cells (Figure 3A, left), which showed that CIP2A was overexpression and partially protected the cells from apoptotic cell death induced by afatinib, and that PP2A activity was decreased (Figure 3B, left). Further, knockdown of the gene expression of CIP2A increased the sensitivity of afatinib-induced apoptosis in the resistant H460 cells (Figure 3A, right) and increased PP2A activity (Figure 3B, right). This suggested that afatinib may repress CIP2A, enhance PP2A activity, and then induce cell death. Furthermore, in order to examine the relationship between PP2A and AKT (Figure 3C), we knocked down PP2Ac expression by siRNA strategy in the sensitive H358 cells. The results showed that AKT phosphorylation increased with the deprived of PP2A and also partially protected the cells from apoptotic death induced by afatinib. In addition, with the treatment of okadaic acid, a known PP2A inhibitor, AKT phosphorylation was significantly increased in the sensitive H358 cells which reduced the afatinib-induced apoptosis (Figure 3D, left), whereas co-treatment with forskolin sensitized resistant H460 cells to afatinib-induced apoptosis and p-AKT downregulation (Figure 3D, right). We next analyzed the role of AKT, which is downstream of PP2A, in mediating the effects of afatinib. The ectopic expression of AKT in the sensitive H358 cells (Figure 3E) partially protected the cells from apoptotic death. Taken together, these results suggest that the afatinib-induced apoptotic effect is through the CIP2A-PP2A-AKT pathway in NSCLC cells.

### Afatinib reduced Elk-1 binding to the CIP2A promoter and suppressed CIP2A transcription

**Figure 4:**
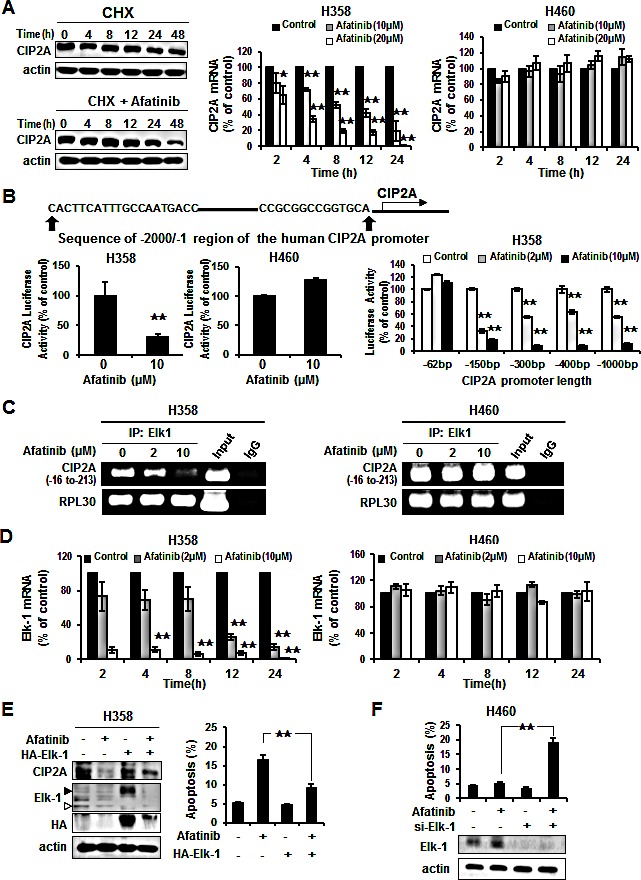
Elk-1 regulated CIP2A in NSCLC cells by afatinib (A) Left, H358 cells were treated with 100 μg/ml cycloheximide (CHX) in the presence or absence of afatinib for the indicated length of time. Middle and Right, H358 and H460 cells treated with afatinib at 10 μM or 20 μM for the designated incubation time. Afatinib inhibited CIP2A mRNA in a dose- and time-dependent manner, especially in H358 cells. Data are mean ± SD. n = 3 for each time point. *, p < 0.05, **, p < 0.01, vs. no afatinib. (B) Effects of afatinib on CIP2A promoter activity. Left, H358 and H460 cells were co-transfected with CIP2A reporter constructs (−1 to −2000bp) and renilla luciferase vectors for 48 h then treated with 10 μM afatinib for an additional 24 h. Afatinib decreased CIP2A luciferase activity in H358 cells, but not in H460 cells. Right, H358 cells were transfected CIP2A reporter various lengths of constructs and renilla luciferase vector for 48 h and then treated with 2 μM or 10 μM afatinib for an additional 24 h. Cell lysates were prepared for analysis of luciferase activity. Data are mean ± SD. n = 3 for each condition. **, p < 0.01, vs. no afatinib. (C) Chromatin immunoprecipitation assays of the CIP2A promoter. H358 and H460 cells were treated with 2 μM or 10 μM afatinib for 24 h and processed for ChIP assay. Soluble chromatin was immunoprecipitated with specific Elk-1 or IgG (negative control) antibodies. Immunoprecipitates were subjected to PCR with primer pair specific to CIP2A promoter (−16 to −213 bp) and RPL30 (internal control). The gel shown is representative of three independent experiments. (D) H358 and H460 cells treated with afatinib at 2 μM or 10 μM for the indicated incubation times. Afatinib inhibited Elk-1 mRNA in a dose- and time-dependent manner, especially in H358 cells. Data are mean ± SD. n = 3 for each time point. **, p < 0.01, vs. no afatinib. (E) Ectopic expression of Elk1 (HA-Elk1) restored the effect of afatinib on CIP2A expression and protected the effect of afatinib-induced apoptosis in H358 cells using Western blotting and FACS. H358 cells overexpressing Elk-1 were treated with 10 μM afatinib for 24 h. Open arrow is endogenous of Elk1 and close arrow is exogenous of Elk1. (F) Knockdown of Elk-1 enhanced apoptosis in H460 cells by afatinib. Protein levels of Elk1 expressed in lower panel. Data are means ± SD. **, p < 0.01.

To examine the mechanism by which afatinib inhibits CIP2A expression, we investigated whether afatinib affected CIP2A protein degradation. After protein translation was blocked by cycloheximide, the rate of CIP2A degradation did not change significantly with or without afatinib in H358 cells (Figure 4A, left). We next investigated whether afatinib affected CIP2A gene transcription. These results showed that the mRNA levels of CIP2A decreased in a time- and dose-dependent manner in the sensitive H358 cells but not in the resistant H460 cells (Figure 4A, right). To further explore the inhibition of CIP2A transcription by afatinib, the sensitive H358 cells were transfected with CIP2A promoter luciferase constructs. Afatinib signiﬁcantly down-regulated the CIP2A promoter activity in a dose-dependent manner in the sensitive H358 cells (Figure 4B). However, afatinib did not alter the luciferase activity in the resistant H460 cells. Among the various lengths of CIP2A promoter regions (-1~ -62 bp, -1~ -150 bp, -1~ -300 bp, -1~ -400 bp, -1~ -1000 bp and -1~ -2000 bp), we observed that each length of the promoters was significantly depressed by increasing the dose of afatinib except for -1~ -62 bp. The putative Elk-1-binding site existed in the -62~ -150 bp element. We therefore performed ChIP assay to evaluate the binding affinity between the transcription factor of Elk-1 and CIP2A promoter. The interaction of Elk-1 with the CIP2A promoter was abolished in a dose-dependent manner in the sensitive H358 cells but not in the resistant H460 cells (Figure 4C). Furthermore, we explored the effect of afatinib on transcription factor of Elk-1, and found that both mRNA (Figure 4D) and protein levels (Figure 4E) of Elk-1 were reduced by afatinib. Moreover, the ectopic expression of Elk-1 also restored CIP2A expression (Figure 4E, left) and partially protected the cells from apoptotic death induced by afatinib in H358 cells (Figure 4E, right). On the other hand, deprivation of Elk-1 increased apoptosis by afatinib in H460 cells (Figure 4F). These results suggest that afatinib may inhibit CIP2A expression by affecting the DNA binding ability of Elk-1 via debasing Elk-1 in the sensitive H358 cells.

### Evaluation of the therapeutic effect of afatinib on H358-bearing mice

**Figure 5:**
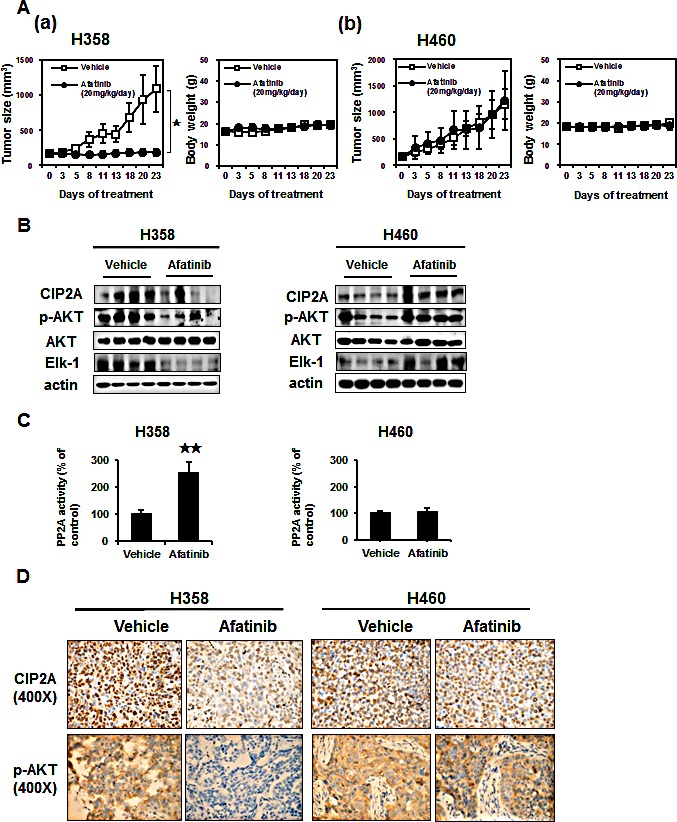
*In vivo* effects of afatinib on xenografts mice (A)(a) Left, effects of afatinib on tumor size in H358 xenografts. Afatinib inhibited the growth of H358 tumors by > 80%. Right, effects on body weight of H358 xenograft mice. (b) Left, effects of afatinib on tumor size in H460 xenograft mice. Right, effects on body weight of H460 xenograft mice. Mice were treated with vehicle, or oral afatinib at 20 mg/kg daily for 3 weeks (n = 8 each). Data are mean ± SD; *, p < 0.05. (B) Western blot analysis of CIP2A, p-AKT and AKT in H358 (Left) and H460 (Right) tumors. (C) The activities of PP2A in H358 (Left) and H460 (Right) tumors. mean ± SD, n = 8 for each condition.**p <0.01. (D) Representative immunohistochemical patterns of CIP2A and p-AKT in xenografts tumors.

To determine whether or not the *in vitro* effects of afatinib on the sensitive H358 cells and the resistant H460 cells could be reproduced *in vivo*, mice were implanted with H358 and H460 xenografts. No apparent differences in body weight or toxicity were found (Figure 5A). Treatment with afatinib significantly inhibited H358 xenografts tumor growth by nearly 80% compared to the controls (Figure 5A-a, left), but not in the H460 xenografts (Figure 5A-b left). To correlate the clinical implications in NSCLC with the mechanism identified *in vitro,* the effects of afatinib on the CIP2A-PP2A-AKT pathway in these tumor were examined by Western blot and PP2A activity assay. Overall, there were significant decreases in CIP2A, p-AKT and Elk-1(Figure 5B) and an enhanced PP2A activity (Figure 5C) in the H358 tumors treated with afatinib (Figure 5B, 5C left), whereas no significant changes were observed in the control (vehicle) or H460 tumors (Figure 5B, 5C right). Immunohistochemical analysis of the tumor specimens demonstrated strong cytoplasmic staining of CIP2A and p-AKT in the vehicles of H358 and H460 cells (Figure 5D). This staining revealed weaker expression under afatinib treatment in the H358 cells (Figure 5D left), but not in the afatinib-resistant H460 cells (Figure 5D right). Taken together, these results confirmed that afatinib increased PP2A activity to repress p-AKT via CIP2A to inhibit tumor growth in this NSCLC xenografts model.

### Increased CIP2A expression in tumor tissues of patients with NSCLC was associated with a poor clinical outcome

**Figure 6:**
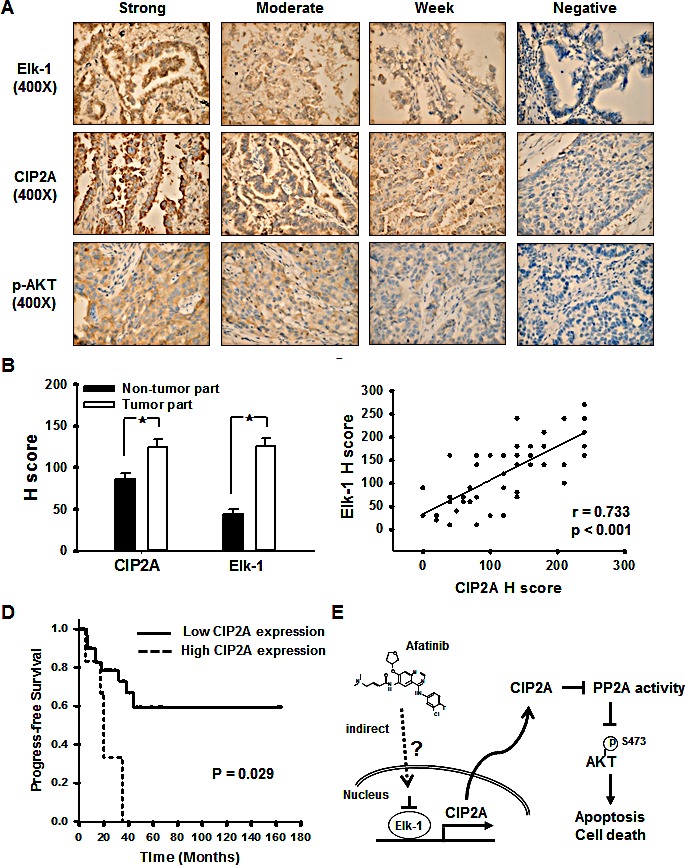
Cancerous inhibitor of protein phosphatase 2A (CIP2A) expression in 49 non-small cell lung cancer patients (A) Strong, moderate, weak, and negative expressions of Elk-1, CIP2A, and p-AKT. (B) Elk-1 and CIP2A expression in the non-tumor part and tumor part; *, p < 0.05. (C) The correlation of Elk-1 and CIP2A, r= 0.773, p <0.001. (D) Kaplan-Meier estimates of progression-free survival in the 49 patients with NSCLC according to high or low CIP2A expression. (E) Schema of the molecular mechanism of afatinib on the Elk-1/CIP2A/PP2A/AKT pathway.

We examined CIP2A and Elk-1 expressions in paraffin-embedded tumor tissues from patients who underwent surgical resection for NSCLC. The clinical characteristics of the patients are shown in Table 1. Immunohistochemistry of the lung cancer tissue sections showed that the expression of CIP2A was frequently observed in the clinical tumor samples from the NSCLC patients and that the expression of CIP2A in the tumor samples was correlated with the expressions of p-AKT and Elk-1 (Figure 6A). Higher CIP2A and Elk-1 expressions were found in the tumor part compared to the non-tumor part (Figure 6B), and the expressions of CIP2A and Elk-1 were highly correlated (r=0.733, *p*<0.001) (Figure 6C). The progression-free survival of the patients with a high CIP2A expression was shorter than for those with a low CIP2A expression (*p*=0.029, log-rank test) (Figure 6D). Taken together, the results suggest that the mechanism of afatinib in sensitive NSCLC cells is by decreasing CIP2A through a reduction in Elk-1 which restores PP2A activity and leads to p-AKT downregulation, thereby inducing cancer-cell apoptosis (Figure 6E).

**TABLE 1:** Clinical Characteristics of patients

Characteristics	CIP2A high	CIP2A low	Total
Number	40	9	49
Gender			
Male	27	4	31
Female	13	5	18
Age (years)			
<65	11	5	16
≥65	29	4	33
Tumor Size			
<3 cm	16	6	22
≥3 cm	24	3	27
Differentiation			
Well	3	1	4
Moderate	25	7	32
Poor	12	1	13
T status			
T1, T2	34	7	41
T3, T4	6	2	8
N status			
N0	31	7	38
N1, N2	9	2	11
Staging			
I, II	32	6	38
III, IV	8	3	11
Histology type			
Squamous cell carcinoma	11	0	11
Adenocarcinoma	29	9	38
Elk-1 expression			
No expression	20	0	20
Expression	20	9	29

## DISCUSSION

In this study, we demonstrated that afatinib-induced apoptosis in NSCLC cell lines without EGFR mutation through Elk-1 mediated CIP2A down-regulation. We identified CIP2A as a major molecular determinant of the apoptosis-inducing effect of afatinib in NSCLC without EGFR mutation. In addition, we found that the overexpression of CIP2A upregulated p-AKT and protected sensitive H358 cells from afatinib-induced apoptosis. Knockdown of the gene expression of CIP2A in the afatinib-resistant H460 cells decreased the phosphorylation of AKT and increased afatinib-induced apoptosis, thus changing the phenotype of the resistant H460 cells to resemble the sensitive H358 cells. Our results and previous studies show that CIP2A is overexpressed in NSCLC and is correlated with a poor prognosis [15, 18, 21]and TNM stage [21]. This strengthens the evidence that CIP2A may be a potential drug target [13, 18, 28-37]. Ma *et al.* demonstrated that Rabdocoetsin B can inhibit proliferation and induce apoptosis in a variety of lung cancer cells by down-regulating CIP2A and inactivating AKT pathway [18]. Our *in vivo* results also showed that afatinib downregulated CIP2A through Elk-1 in H358 xenograft tumors and inhibited tumor growth (Figure 5). These structurally unrelated agents showed a common target in different cancer cells, suggesting that CIP2A may be a novel anti-cancer target.

Elk-1 is a member of the ETS oncogene family. They are transcription factors involved in many biological processes, including cell growth and survival, angiogenesis, wound healing and cancer [18, 38]. The ETS transcription factor family is defined by the presence of a highly conserved DNA-binding domain, and the proto-oncogene c-fos characterized as an Elk-1 target [39]. Promoter activity is regulated by the serum response element (SRE), and recruitment of Elk-1 to the SRE is through the combination of protein-protein or protein-DNA interactions [40]. Pallai *et al.* demonstrated that the binding of Ets-1 and Elk-1 together to the proximal CIP2A promoter is required for CIP2A expression in cervical, endometrial and liver carcinoma cell lines [41]. However, another study showed that only Ets-1 is required to regulate CIP2A expression in prostate and gastric carcinoma [42]. Our ChIP experiments using an Elk-1 specific antibody revealed that afatinib reduced CIP2A promoter occupancy by Elk-1 in a dose-dependent manner in sensitive H358 cells but not in resistant H460 cells (Figure 4C). Afatinib did not change the CIP2A promoter occupancy by Ets-1 (data not shown). Thus, in NSCLC treated with afatinib, Elk-1 mediates CIP2A expressed reduction causing of interfered with the function of Elk-1. Addition, the Raf-1/MEK/ERK signaling cascades activated downstream transcription factor Elk-1 has been proved which involved in Elk-1-regulated EMT [43]. In order to verify how Elk-1 is modulated, we have additionally performed experiments with knockdown of ErbB family or KRAS (Supplement Figure S1). There are no apparently alterations of Elk-1 under HER2 siRNA, Erb4 siRNA, KRAS siRNA, or EGFR siRNA. Therefore, afatinib may modulate Elk-1 not through ErbB family or KRAS. Furthermore, we have additionally performed experiments to see the change of CIP2A after adding afatinib under different EGFR status (Supplement Figure S2). In H358 (EGFR wild) and PC9 (EGFR mutation), we tested the effect of afatinib or EGFR siRNA. Under EGFR siRNA only (without afatinib), EGFR decreased but CIP2A did not show any change. Adding afatinib and EGFR siRNA together, CIP2A decreased. Therefore the decrement of CIP2A may be not through EGFR, no matter in EGFR wild or EGFR mutation NSCLC cell lines.

Clinical trials for afatinib, such as LUX-Lung 3 [26] and LUX-Lung 6 [25], have focused on NSCLC patients harboring EGFR mutations. However, NSCLC patients without EGFR mutation are still a big population in NSCLC [44]. Current, there is a phase II study of afatinib as third-line treatment for patients in Korea with stage IIIB/IV non-small cell lung cancer harboring wild-type EGFR [45]. In this trial, 42 patients received afatinib treatment but then 38 of those were included in efficacy analyses (other 4 patients were tested positive for EGFR mutations later). No complete response or partial responses were found and disease control rate was 24% with median disease control duration of 19.3 weeks. In addition, several studies on other TKIs have shown effects in NSCLC patients without EGFR mutation. In the phase 3 Sequential Tarceva in Unresectable NSCLC (SATURN) trial, erlotinib was used as a second-line NSCLC maintenance therapy and showed a better progression-free survival than placebo in the patients without EGFR mutation (hazard ratio 0.78, 95% CI 0.63–0.96; *p*= 0.0185) [10]. In the Tarceva In Treatment of Advanced NSCLC (TITAN) study, there was no significant difference in the patients treated with second-line erlotinib and those treated with docetaxel or pemetrexed in the subgroup without EGFR mutation (hazard ratio 0.85, 95% CI 0.59–1.22; *p*= 0.37) [11]. Therefore, afatinib may be effective in patients without EGFR mutation. Our results demonstrate a possible mechanism and suggest a potential biomarker for the selection of suitable NSCLC patients for future afatinib trials.

Besides, afatinib may also effective in patients with acquired resistance to erlotinib or gefitinib. In the animal study, the combine use of afatinib and cetuximab, the EGFR-specific antibody, can dramatic shrinkage of erlotinib-resistant tumors harboring the T790M mutation [46], There are also the clinical trials in the combination of afatinib and cetuximab for *EGFR*-mutant lung cancers with acquired resistance to gefitinib or erlotinib, both with and without *T790M* mutations. Among 126 patients, objective response rate (overall 29%) was comparable in *T790M*-positive and *T790M*-negative tumors (32% vs. 25%; *P*= 0.341). Median progression-free survival was 4.7 months (95% confidence interval, 4.3–6.4); median duration of confirmed objective response was 5.7 months (range, 1.8–24.4)[47]. There may be other pathways besides dual inhibition of EGFR. Our results may offer other mechanism to explain the possibility of combinations of afatinib and cetuximab.

In conclusion, afatinib induced apoptosis in NSCLC cell lines without EGFR mutation through a novel mechanism, the Elk-1/CIP2A/PP2A/p-AKT pathway. CIP2A was a major molecular determinant of the sensitivity of NSCLC without EGFR mutation to afatinib-induced apoptosis. Focusing on the interactions of oncoproteins, phosphatases and kinases could be a novel anti-cancer strategy. Future studies to elucidate the mechanism by which afatinib inhibits Elk-1 may lead to further progress in the development of molecular-targeted therapy for lung cancer.

## METHODS

### Patient samples collection

The Institutional Review Board of Cardinal Tien Hospital (CTH) approved the study protocol. Tumor specimens from 49 patients who underwent surgical resection for primary NSCLC at CTH between 2004 and 2009 were examined. All clinical charts and histopathology reports were reviewed for data on age, gender, site, diagnosis, differentiation, tumor size, lymph node or distant metastasis, and TMN staging. All patients were followed with the disease status of disease free or disease progression. At the end of current study, survival status of each subjects were also recorded.

All tissue samples were routinely fixed in formalin and embedded in paraffin wax. Representative tissue areas were chosen at the junction of the major tumor mass and the adjacent benign area marked on standard hematoxylin and eosin (H&E) sections taken from the paraffin block using a 2.0-mm punch, and inserted into a recipient paraffin block. Four-μm-thick sections were cut from the completed array block and transferred to silanized glass slides.

### Histology, immunohistochemistry and scoring

The constructed tissue array paraffin embedded blocks were cut into 5-μm-thick sections for H&E staining. For each case, carcinoma type, cell differentiation, growth pattern, tumor cell nuclear morphology, metaplasia, calcification, necrosis, mitosis count, invasion status and other specific differentiations were re-checked by two pathologists. Immunohistochemical (IHC) staining was performed using a Ventana BenchMark XT automated stainer (Ventana, Tucson, AZ). Briefly, 4-μm-thick sections were cut consecutively from formalin-fixed, paraffin-embedded tissue. These sections were then mounted on silanized slides and allowed to dry overnight at 37°C. After deparaffinization and rehydratation, the slides were incubated with 3% hydrogen peroxide solution for 5 minutes. After washing with the supplied buffer, the tissue sections were repaired for 40 minutes with ethylenediamine tetraacetic acid.

The slides were then incubated with the primary antibodies overnight at 4°C, including, anti-p-AKT1(Ser473) (1:50, Genetex, Irvine, CA), anti-CIP2A (1:50, *Novus* Biologicals, Littleton, CO), anti-Elk-1 (1:25, Santa Cruz, San Diego, CA). After three rinses in buffer solution, the slides were then incubated with the secondary antibodies (unbiotinylated antibody, EnVisionTM System, HRP, anti-mouse/rabbit, DakoCytomation). Tissue staining was visualized using DAB substrate chromogen solution (DakoCytomation). The slides were counter-stained with hematoxylin, dehydrated, and mounted. Each run included using phosphate buffered solution as the primary antibody for the negative control, and the samples known to express these markers strongly served as the positive control. Two experienced pathologists reviewed the immunohistochemical slides, and immunostaining intensity results were recorded as 0 for no staining, 1 for faint, 2 for moderate, and 3 for intense staining. The percentage of staining of each core was also recorded from 0% to 100%. The H-score (from 0 to 300) was calculated by multiplying the staining intensity and percentage of each core. The immunostaining results were classified as negative for an H-score of 100 or less, and positive for an H-score of more than 100.

### Cell culture

Four NSCLC cell lines were used in this study. H358 (bronchioloalveolar carcinoma [BAC], mutant KRAS), H441 (papillary adenocarcinoma, mutant KRAS, and TP53), and A549 (BAC, mutant KRAS, CDKN2A, and STK11) cell lines were obtained from the American Type Culture Collection (Manassas, VA) and the H460 (large cell lung cancer, mutant KRAS, PIK3CA, STK11, and CDKN2A) cell line was obtained from the Bioresource Collection and Research Center (Hsinchu, Taiwan). The NSCLC cell lines were kept in RPMI1640 (Invitrogen, Life Technologies, Saint Aubin, France) supplemented with 10% FBS (GIBO/Life Technologies, Grand Island, NY), 100 units/mL penicillin G and 100 μg/mL streptomycin sulfate in a 37°C humidified incubator with 5% CO_2_ in air.

### Reagents and antibodies

Afatinib (Giotrif®) was purchased from Selleck chemicals (Houston, TX). For *in vitro* studies, afatinib at various concentrations was dissolved in DMSO and then added to cells in serum-free RPMI1640. PP2A inhibitor and activator were purchased from Sigma (*Sigma*-Aldrich, St. Louis, Missouri) and Merck Millipore (Billerica, MA), respectively. Antibodies for immunoblotting including anti-CIP2A, AKT, Elk-1 and PARP were purchased from Santa Cruz Biotechnology (San Diego, CA). Other antibodies including anti-PP2A and p-AKT (Ser473) were purchased from Cell Signaling (Danvers, MA).

### Apoptosis analysis and caspase-3 activity assay

Apoptotic cells were measured by flow cytometry (sub-G1 analysis) and drug-induced apoptotic cell death was detected by Western blot analysis of PARP cleavage. Apoptotic DNA fragmentation was measured using a Cellular DNA fragmentation ELISA kit (Roche, Diagnostics Corp, Indy) according to the manufacturer's instructions. The activity of caspase-3 was determined using a caspase-3 assay kit (Abcam Inc., Cambridge, MA), which detected the cleavage of chromogenic caspase-3 substrates (Ac-DEVD-*p*NA). Protein was extracted using ice-cold cell lysis buffer, and total protein (1–3 mg/ml) was added to the reaction buffer containing 10 ul Ac-DEVD-*p*NA (2 mM), followed by incubation for 60–120 minutes at 37°C. The free *p*NA cleaved from its precursor was quantified using a spectrometer at 405 nm. A comparison of the absorbance of *p*NA from an apoptotic sample with an un-induced control allowed for the determination of the fold increase in caspase-3 activity.

### Ectopic expression of CIP2A, AKT or Elk-1 and cell transfection

CIP2A cDNA (KIAA1524), AKT1 cDNA and Elk-1 cDNA were purchased from OriGene (Rockville, MD). Briefly, following transfection, H358 cells were incubated in the presence of G418 (0.78 mg/mL) (Sigma-Aldrich; St. Louis, MO). After 8 weeks of selection, surviving colonies, i.e., those arising from stably transfected cells were selected and individually amplified. The H358 cells that were transient transfected with Elk-1, CIP2A or AKT1 were then treated with afatinib, harvested, and processed for Western blot analysis. Transfection was carried out the X-tremeGENE HP Transfection *Reagent* according to the manufacturer's instructions (Roche, Diagnostics Corp, IN).

### PP2A phosphatase Activity

Protein phosphatase 2A (PP2A) activity was measured in fresh cells as described previously using an R&D Systems PP2A DuoSet IC activity assay kit according to the manufacturer's instructions (R&D Systems, Minneapolis, MN). Briefly, an immobilized capture antibody specific for the catalytic subunit of PP2A that binds both active and inactive PP2A was used. After washing, a substrate was added that is dephosphorylated by active PP2A to generate free phosphate, which is then detected by a sensitive dye-binding assay using malachite green and molybdic acid.

### Gene knockdown using siRNA

Smart-pool siRNA, including control (sc-37007), CIP2A and PP2A were purchased from Santa Cruz Biotechnology (San Diego, CA). Cells were transfected with siRNA to a final concentration of 100 nM in 6-well plates with Dharma-FECT4 transfection reagent (Dharmacon, Chicago, IL). After 48 hours, the medium was replaced and the cancer cells were harvested for analysis by Western blot and flow cytometry to measure apoptosis.

### Quantification of CIP2A and Elk-1 gene expressions

Total RNA was extracted from H358 and H460 cells (approximately 5 × 10^6^ ) followed by afatinib treatment using an RNeasy Mini Kit (Qiagen, Gaithersburg, MD), and then reverse transcribed using a QuantiTect Reverse Transcription kit (Qiagen, Gaithersburg, MD). The real time quantitative PCR was performed on an Applied Roter-Gene 3000 detector (Qiagen, Gaithersburg, MD) with a specific primer set for each target gene and SYBR Green dye (Qiagen, Gaithersburg, MD) according to the manufacturer's instructions. The PCR primer sets for the target genes were as follows: human CIP2A (Hs_KIAA1524 QuantiTect Primer Assay (NM_020890)), human Elk-1 (Hs_Elk-1 QuantiTect Primer Assay (NM_005229)) and human actin (Hs_ACTB QuantiTect Primer Assay (NM_001101)). An aliquot of each sample was analyzed by quantitative PCR for actin to normalize for inefficiencies in cDNA synthesis and the amount of RNA input. For each sample, the average threshold (Ct) value was determined from quadruplicate assays, and the ΔCt value was determined by subtracting the average actin Ct value from the average CIP2A Ct value. Three independent experiments were performed to measure the levels of CIP2A and Elk-1 of H358 and H460 cells with different durations of treatment.

### Chromatin immunoprecipitation

Chromatin immunoprecipitation was performed using 1-2 × 10^7^ H358 or H460 cells. The cells were treated with DMSO or afatinib for 24 hours. After afatinib treatment, the cells were treated with 1% formaldehyde to crosslink for 10 min at room temperature, by adding 1X glycine to stop crosslink. Then cells were then washed twice with ice-cold PBS and scraped in ice-cold PBS containing protease inhibitors (Roche, Diagnostics Corp, IN). The cells were then collected and washed twice with PBS followed by centrifugation at 700x*g* for 5 minutes at 4°C. The cell pellets were lysed in ice-cold RIPA buffer with protease inhibitors, and chromatin was sheared via sonication on a cup horn for 6 minutes with 30-second bursts to yield an average DNA fragment length of approximately 100-1000 base pairs. Lysates were clarified by centrifugation at 12,500x*g* for 5 minutes at 4°C, diluted 1:5 with chromatin immunoprecipitation (ChIP) dilution buffer (Novus Biologicals, Inc., Littleton, CO), and incubated overnight at 4°C with the following antibodies: anti-Elk-1 (Abcam Inc., Cambridge, MA) or mouse IgG (Invitrogen, Life Technologies, Grand Island, NY) as a control. Immunoprecipitation and DNA purification were performed according to manufacturer's protocol (ChromataChIP Kit, Novus Biologicals, Inc., Littleton, CO). The isolated DNA was used for quantitative PCR.

### Dual-luciferase reporter assay

To verify the transcriptional activity between the erlotinib-sensitive (H358) and erlotinib-resistant (H460) cells, the promoter activity of *CIP2A* was determined using a dual-luciferase reporter assay kit (Promega, Madison, WI). The H358 and H460 cells were co-transfected in 6-well plates with 2 μg of DNA, including the luciferase reporter construct pGL4.17-*CIP2A*-promoter, and pRL-*TK* vectors (Promega, Madison, WI) as an indicator for the normalization of transfection efficiency, at a ratio of 9:1. Forty-eight hours post-transfection, various doses of afatinib were added. After twenty-four hours, the cells lysates were collected and luciferase activity was quantified according to the manufacturer's instructions (Promega, Madison, WI). Cells co-transfected with pGL4.17-basic plasmid (Promega, Madison, WI) combined with the pRL-*TK* plasmid were used as a negative control. The promoter activity was repeated three times in parallel for statistical analysis.

### Xenograft tumor growth

Male NCr nude mice (5-7 weeks of age) were used and all experimental procedures were performed according to protocols approved by the Institutional Laboratory Animal Care and Use Committee of Cardinal Tien Hospital. Each mouse was inoculated subcutaneously in the dorsal flank with 1 × 10^7^ H358 or H460 cells suspended in 0.1 ml of serum-free medium containing 50% matrigel (BD Biosciences, Bedford, MA). When the tumors reached 100-200 mm^3^, the mice were given afatinib (20 mg/kg) p.o. once daily. The controls received vehicle. The tumors were measured twice weekly using calipers and their volumes were calculated using the following standard formula: width x length x height x 0.523 [36].

### Statistical analysis

Statistical analysis was performed using a two-tailed Student's *t*-test. The results were expressed as mean ± standard deviation (SD). Differences were considered significant at *p*<0.05 and highly significant at *p*<0.01.

## SUPPLEMENTARY MATERIAL, FIGURES, TABLES


