# Supplementary material for: Afatinib induces apoptosis in NSCLC without EGFR mutation through Elk-1-mediated suppression of CIP2A
Source: Oncotarget. 2014 Dec 11;6(4):2164–79. doi: 10.18632/oncotarget.2941 (PMC4385843; doi:10.18632/oncotarget.2941)
Supplement: Supplementary file 1 [file oncotarget-06-2164-s001.pdf]

Afatinib induces apoptosis in NSCLC without EGFR mutation through Elk-1-mediated suppression of CIP2A

Supplementary Material

Table

Charateristic of the NSCLC cell lines used in this study with different mutation status[a].

Cell line	Subtype	*EGFR*	*HER2*	*KRAS*	*BRAF*	*PIK3CA*	*TP53*	*PTEN*
A549	Adenocarcinoma	Wild	Wild	G12S	Wild	Wild	Wild	Wild
H460	Large cell carcinoma	Wild	Wild	Q61H	Wild	E545K	Wild	Wild
H358	Adenocarcinoma	Wild	Wild	G12C	Wild	Wild	Null	Wild
H441	Adenocarcinoma	Wild	Wild	G12V	Wild	Wild	R158L	Wild

Abbreviations: EGFR, epidermal growth factor receptor; *HER2*, human epidermal growth factor receptor 2; *KRAS*, Kirsten rat sarcoma viral oncogene homolog; *BRAF*, *PIK3A*, phosphoinositide-3-kinase, catalytic, alpha polypeptide; *TP53*, tumor protein *p53*; *PTEN*, phosphatase and tensin homolog.

[a] This table was modified from Rikova K *et al.* (*Cell* 2007; 131:1190-1203)

Supplemental Data

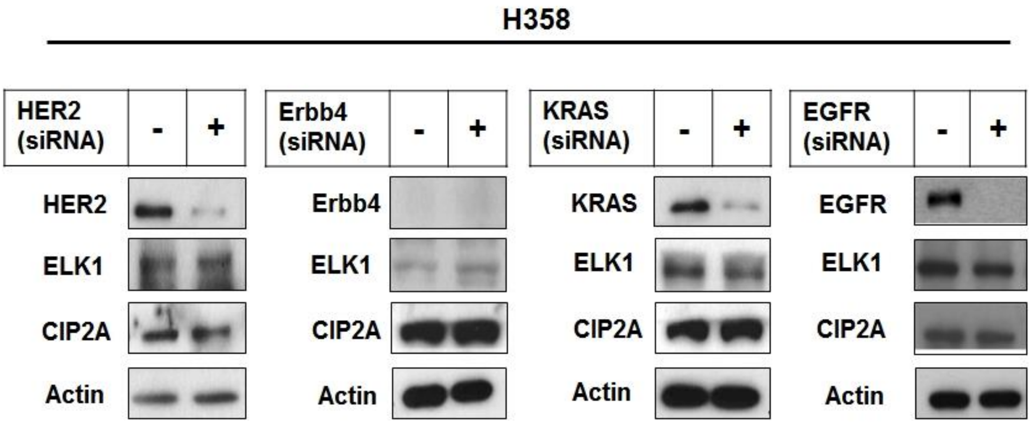

Figure S1: Knockdown of ErbB family and KRAS genes in afatinib-sensitive (H358) cells.

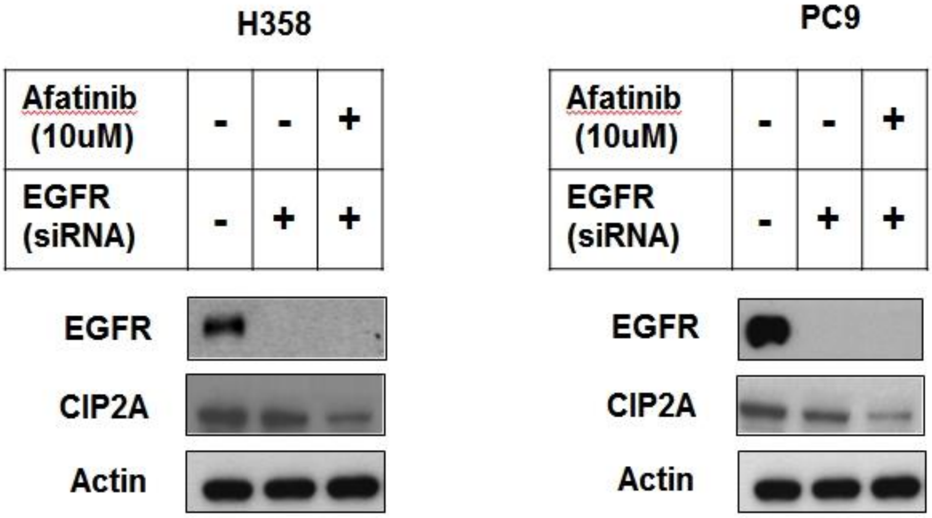

Figure S2: Knockdown of EGFR and combines with afatinib in afatinib-sensitive (H358) cells and PC9 (EGFR mutation) cells.
